# Infectious Endocarditis Accompanied by Leukocytoclastic Vasculitis

**DOI:** 10.7759/cureus.61021

**Published:** 2024-05-24

**Authors:** Joshua Lunsford, Prajwol Pant, Patrick S Rush

**Affiliations:** 1 Internal Medicine, Edward Via College of Osteopathic Medicine, Blacksburg, USA; 2 Nephrology, Blue Ridge Nephrology Associates, Christiansburg, USA; 3 Nephrology, Carilion New River Valley Hospital, Christiansburg, USA; 4 Dermatology, Virginia Tech Carilion School of Medicine, Roanoke, USA; 5 Dermatology, Dominion Pathology Associates, Roanoke, USA

**Keywords:** aortic valve vegetation, leukocytoclastic vasculitis (lcv), purpura, endocarditis, cutaneous vasculitis

## Abstract

Bacterial endocarditis is a rare infection that can present with variable clinical manifestations. Rarely, it can present as cutaneous vasculitis characterized by a purpuric rash mimicking immune-mediated vasculitis. There have been a few case reports of leukocytoclastic vasculitis (LCV) due to infectious endocarditis. It is important to recognize endocarditis as a potential cause of vasculitis because treatment with immunosuppressive agents can have devastating consequences. We report a case of a 53-year-old male with endocarditis who developed a palpable purpura of the bilateral lower extremities. A skin biopsy was performed, and histopathologic and immunofluorescence studies demonstrated LCV.

## Introduction

Endocarditis refers to inflammation of the endocardium and the heart valves and is typically caused by a bacterial infection. It is a rare condition with an estimated prevalence of 3 to 10 cases per 100,000 people [1]. The most commonly isolated organisms are Gram-positive staphylococci, streptococci, and enterococci. Less commonly implicated organisms include the HACEK organisms (*Haemophilus*, *Actinobacillus*, *Cardiobacterium*, *Eikenella*, and *Kingella*). Risk factors include intravenous drug use, immunosuppression, poor dentition, degenerative valvular disease, and rheumatic heart disease [2]. Clinical presentation is variable and usually consists of a constellation of nonspecific symptoms, including the insidious onset of fevers, chills, fatigue, and malaise [1]. The classic physical exam finding is the presence of a new or worsening heart murmur; however, this occurs in less than 50% of patients [2]. Dermatologic manifestations include Osler nodes, Janeway lesions, and splinter hemorrhages. These findings are well documented [3-5], but they only occur in a minority of patients. Rarely, endocarditis can cause a cutaneous vasculitis that mimics immune-mediated vasculitis [6]. We report a case of endocarditis accompanied by leukocytoclastic vasculitis (LCV) to raise awareness of this potential manifestation and to promote timely treatment with antibiotic therapy.

## Case presentation

A 53-year-old male with a past medical history of type 2 diabetes mellitus, chronic thrombocytopenia, liver cirrhosis, hepatitis B, hepatitis C, hypothyroidism, gastroesophageal reflux disease, depression, and recent pulmonary embolism treated with apixaban presented to the hospital due to one month of dark urine and dark, tarry stools. A workup for gastrointestinal bleeding was performed and was negative.

The patient also reported a history of intermittent fevers for the last few months. Blood cultures were positive for *Streptococcus gordonii*. Transthoracic echocardiography revealed 1-centimeter (cm) aortic valve vegetation. A transesophageal echocardiogram revealed a 1.15 x 0.46 cm aortic valve mass with severe aortic regurgitation. Cardiothoracic surgery was consulted, but it was felt that the patient was not a surgical candidate due to his multiple comorbidities. Infectious disease was consulted and recommended treatment with IV ceftriaxone.

**Figure 1:**
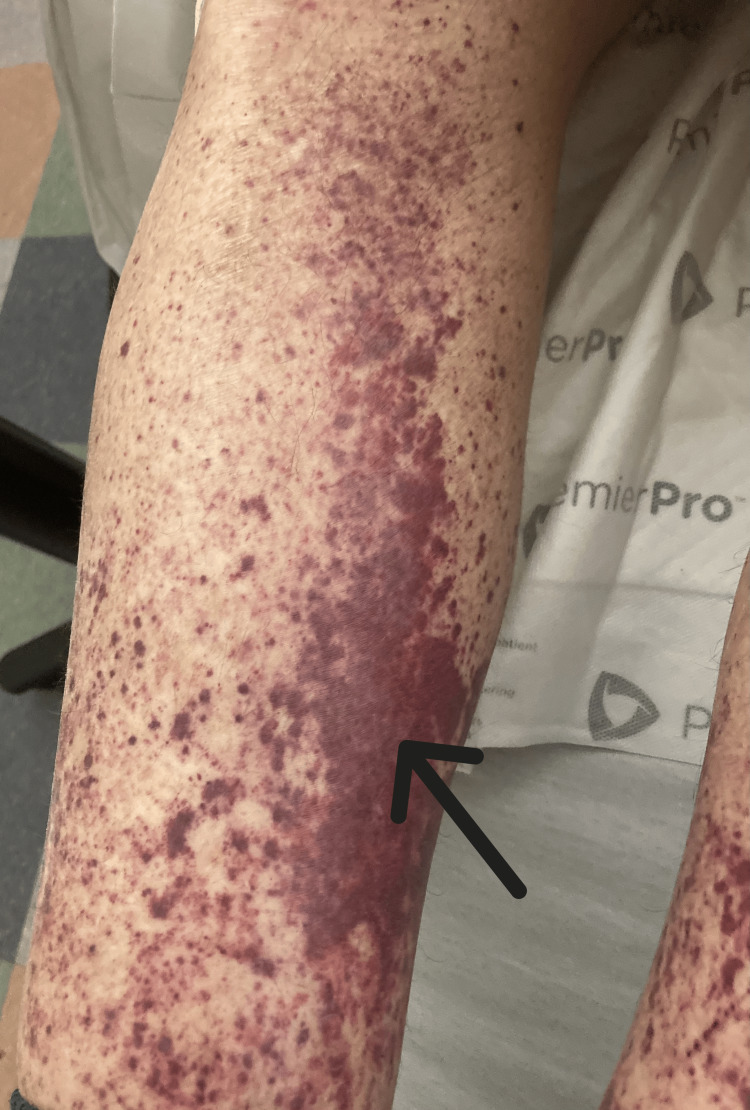
Unilateral view of purpuric rash coalescing into purpuric plaques

**Figure 2:**
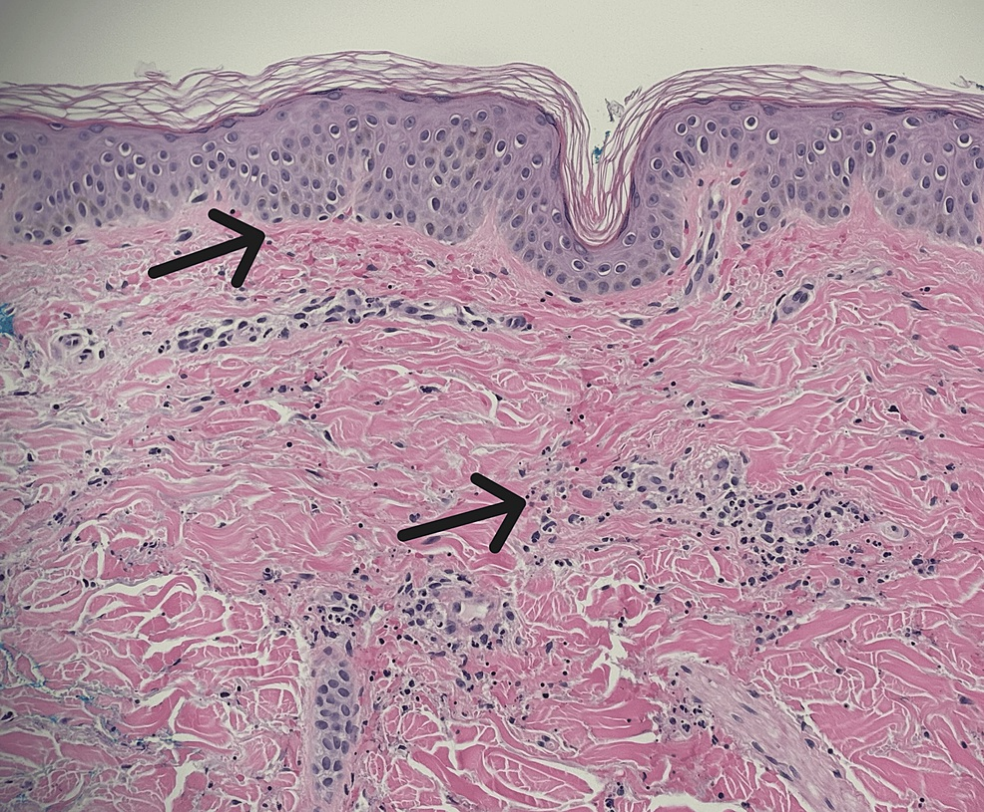
Medium power (20x) H&E showing extravasated red blood cells (top arrow), and regions of leukocytoclasia (bottom arrow) Leukocytoclasia: a process where neutrophils break down and release debris H&E: hematoxylin and eosin

**Figure 3:**
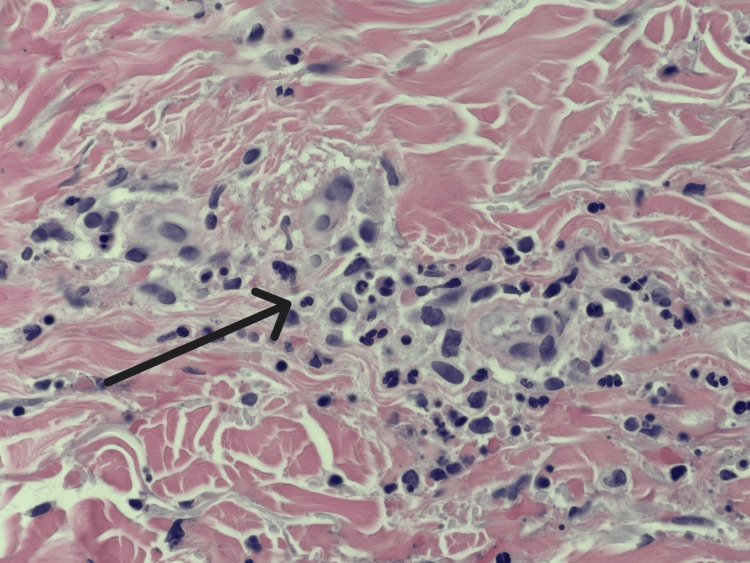
High power (60x) H&E showing clear areas of vascular damage by neutrophils with fibrin deposition within the vessel wall of small vessels H&E: hematoxylin and eosin

**Figure 4:**
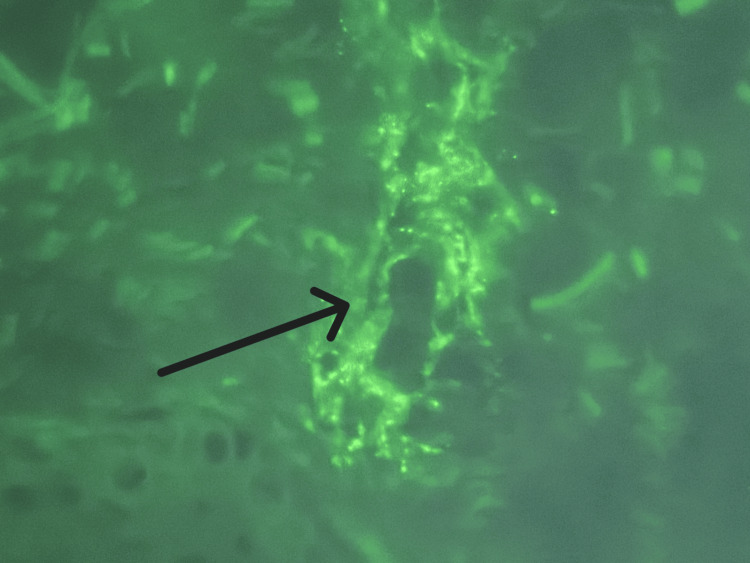
60x direct immunofluorescence IgM showing strong 3+ granular perivascular deposition (C3 (not pictured) showed a similar pattern) 3+: refers to the strength of deposition

The patient developed a palpable purpura of his bilateral lower extremities (Figure 1), which was initially thought to be due to his chronic thrombocytopenia, but his platelets were stable at >100 k/uL (130-400). A spot urine with urine protein-to-creatinine ratio was significant for protein-to-creatinine ratio >200 mg/g. Antinuclear antibody (ANA), perinuclear antineutrophilic cytoplasmic antibody (p-ANCA), antineutrophilic cytoplasmic antibody (c-ANCA), serum immunoglobulins, C3, C4, and cryoglobulins were checked due to concern for vasculitis. Significant findings included IgA 754 mg/dL (47-310), C3 50.8 mg/dL (90-170), and C4 <10 mg/dL (12-36). ANA, p-ANCA, c-ANCA, IgM, IgG, and cryoglobulins were within normal limits. Rheumatology was consulted and recommended a skin biopsy to look for LCV. Dermatology was consulted, and three 4 mm punch biopsies were performed. Histopathology and immunofluorescence studies were performed and demonstrated IgM, fibrinogen, and C3 perivascular deposition (Figures 2-4). Due to the histopathology and immunofluorescence studies demonstrating immunocomplex deposition and the patient's low serum complement levels suggesting complement consumption, the patient was diagnosed with LCV. Given the patient's history of hepatitis B and C and the known association of these conditions with LCV, hepatitis C virus (HCV) ribonucleic acid (RNA), and hepatitis B surface antigen (HBsAg) were checked and were both negative.

The patient’s hospital course was complicated by decompensated cirrhosis, spontaneous bacterial peritonitis, renal failure requiring continuous renal replacement therapy/hemodialysis, and respiratory failure requiring intubation. He was ultimately placed on comfort care due to a low likelihood of clinical improvement.

## Discussion

Dermatologic manifestations of infectious endocarditis are well-documented and classically consist of Osler’s nodes, Janeway lesions, and splinter hemorrhages [3-5]. Less commonly, patients may develop LCV. LCV is a cutaneous, small-vessel vasculitis of the dermal capillaries and venules [7]. It is most commonly idiopathic but may be caused by drugs, infections, neoplasms, and autoimmune disorders [7]. Rarely, LCV can be the presenting symptom in a patient with a systemic bacterial infection such as infectious endocarditis. In 2015, J. Loricera et al. [6] assessed the presentation of cutaneous vasculitis with systemic bacterial infection by evaluating 766 patients diagnosed with cutaneous vasculitis. Out of those assessed, 27 were found to have a systemic bacterial infection, and six of those were diagnosed with infectious endocarditis. Various cases have described patients initially presenting with lower extremity purpuric rash and eventually being diagnosed with bacterial endocarditis [8-12]. Additionally, endocarditis has been shown to mimic other vasculitis-causing conditions such as HSP, ANCA-vasculitis, and cryoglobulinemia [12-15]. It is important to keep infectious causes of vasculitis in the differential diagnosis when evaluating patients presenting with a purpuric rash because treatment for infection-related vasculitis differs significantly from that of primary autoimmune vasculitis. Treatment for primary autoimmune vasculitis consists of corticosteroids and other immunosuppressive agents such as cyclophosphamide, rituximab, azathioprine, and TNF-alpha inhibitors whereas treatment for infection-related vasculitis is aimed at treating the underlying infection. Treating infection-related vasculitis with immunosuppressants can result in detrimental adverse effects, clinical deterioration, and even death. Wang et al. [15] reported a case of an 87-year-old male who presented with general fatigue and decreased appetite. He was found to have a positive p-ANCA and was diagnosed with p-ANCA-associated vasculitis. He was subsequently treated with steroid pulse therapy, which resulted in an initial improvement in his symptoms. However, he worsened about one week after initiation of treatment and died. An autopsy was performed and showed bacterial vegetation attached to the aortic valve. On the other hand, treatment of the endocarditis with appropriate antibiotics usually leads to resolution of the vasculitis [8-12]. Unfortunately, our patient’s case was complicated by significant comorbidities ultimately leading to his death, which prevented us from evaluating the resolution of his LCV with treatment of the bacterial endocarditis. However, based on his confirmed case of endocarditis and LCV along with the absence of positive antibodies to suggest primary autoimmune vasculitis, his symptoms most likely would have resolved with successful treatment of his endocarditis. Due to the differing treatment strategies and the potential for devastating consequences with improper treatment, it is imperative that infectious etiologies be considered in patients presenting with symptoms of vasculitis. This is especially true in patients with risk factors for systemic infection such as those with immunosuppressing conditions, intravenous drug users, and those with artificial heart valves. Along with preexisting literature (Table 1), our case serves to highlight the association between infectious endocarditis and cutaneous vasculitis.

**Table 1:** Reported cases of LCV associated with endocarditis MSSA: methicillin-sensitive *Staphylococcus aureus*; HSP: Henoch-Schönlein purpura; LCV: leukocytoclastic vasculitis

Study	Case Summary
El Chami et al. 2017 [8]	A female in her 40s presented with night sweats and a non-blanching purpuric rash on her bilateral lower extremities. A transesophageal echocardiogram showed a 1.6 cm vegetation on the aortic valve. A skin biopsy showed leukocytoclastic vasculitis. The rash resolved with antibiotic treatment.
Tous-Romero et al. 2017 [9]	A male in his 40s presented with a three-week history of palpable purpura on the bilateral lower extremities and an eight-month history of fever, asthenia, and weight loss. Blood cultures were positive for *Streptococcus gallolyticus*. Echocardiogram was positive for vegetation affecting the mitral, aortic, and tricuspid valves. A skin biopsy revealed leukocytoclastic vasculitis. Heart valve replacement was performed, and his symptoms resolved.
Spindel et al. 2021 [11]	A female in her 70s with MSSA bacteremia was found to have cutaneous and renal leukocytoclastic vasculitis. An echocardiogram revealed vegetation near her right atrial pacemaker lead consistent with infectious endocarditis. The vasculitis resolved with antibiotic therapy.
Salahuddin et al. 2015 [10]	A female in her 20s presented one month after a dental extraction with a painful purpuric rash on her bilateral lower extremities. Blood cultures were positive for *Staphylococcus epidermidis*. A transesophageal echocardiogram revealed a large mitral valve vegetation. Skin biopsy was consistent with leukocytoclastic vasculitis.
Natajaran et al. 2012 [12]	A male in his 50s presented with a purpuric rash on his bilateral lower extremities as well as a two-month history of fatigue and weight loss. The initial diagnosis was HSP, however, a skin biopsy revealed leukocytoclastic vasculitis. Blood cultures were positive for *Enterococcus faecalis*­, and an echocardiogram showed a large aortic valve vegetation consistent with endocarditis. The rash resolved with antibiotic therapy.

## Conclusions

Although infectious endocarditis is a rare cause of LCV, it should be included in the differential diagnosis of patients with signs and symptoms of cutaneous vasculitis. This is especially true for patients at increased risk of infectious endocarditis. A high index of suspicion is required for proper diagnosis and treatment, which can prevent catastrophic patient outcomes.

## References

[1] Cahill TJ, Prendergast BD (2016). Infective endocarditis. Lancet.

[2] Yallowitz AW, Decker LC (2024). Infectious endocarditis. StatPearls [Internet].

[3] Hirai T, Koster M (2013). Osler's nodes, Janeway lesions and splinter haemorrhages. BMJ Case Rep.

[4] Gogos C, Moschovidis V, Adamopoulos C, Trigoni A, Styliadis I, Sachpekidis V (2021). A case series of skin manifestations of infective endocarditis in contemporary era: just another book finding or a useful clinical sign?. Eur Heart J Case Rep.

[5] Bernardes Filho F, Machado CC, Queiroz RM, Nery B (2018). Hallmark cutaneous signs of infective endocarditis. J Emerg Med.

[6] Loricera J, Blanco R, Hernández JL (2015). Cutaneous vasculitis associated with severe bacterial infections. A study of 27 patients from a series of 766 cutaneous vasculitis. Clin Exp Rheumatol.

[7] Baigrie D, Goyal A, Crane JS (2024). Leukocytoclastic vasculitis. StatPearls [Internet].

[8] El Chami S, Jibbe A, Shahouri S (2017). Bacterial endocarditis presenting as leukocytoclastic vasculitis. Cureus.

[9] Tous-Romero F, Delgado-Márquez AM, Gargallo-Moneva V, Zarco-Olivo C (2017). Cutaneous vasculitis: a presentation with endocarditis to keep in mind. An Bras Dermatol.

[10] Salahuddin H, Luni FK, Siddiqui N, Rohs M, Kaw D, Altorok N (2015). Bacterial endocarditis complicated by leukocytoclastic vasculitis. Am J Med Sci.

[11] Spindel J, Parikh I, Terry M, Cavallazzi R (2021). Leucocytoclastic vasculitis due to acute bacterial endocarditis resolves with antibiotics. BMJ Case Rep.

[12] Natarajan A, Hindocha D, Kular N, Fergey S, Davis JR (2012). Vasculitic rash: do not jump to conclusions. Clin Med (Lond).

[13] Fukasawa H, Hayashi M, Kinoshita N (2012). Rapidly progressive glomerulonephritis associated with PR3-ANCA positive subacute bacterial endocarditis. Intern Med.

[14] Agarwal A, Clements J, Sedmak DD, Imler D, Nahman NS Jr, Orsinelli DA, Hebert LA (1997). Subacute bacterial endocarditis masquerading as type III essential mixed cryoglobulinemia. J Am Soc Nephrol.

[15] Wang KY, Shimajiri S, Yoshida T, Yamada S, Sasaguri Y (2010). An autopsy case of microscopic polyangiitis associated with bacterial endocarditis (Article in Japanese). J UOEH.

